# Supplementary material for: The Spatial Distribution Patterns, Physicochemical Properties, and Structural Characterization of Proteins in Oysters (Crassostrea hongkongensis)
Source: Foods. 2022 Sep 13;11(18):2820. doi: 10.3390/foods11182820 (PMC9497732; doi:10.3390/foods11182820)
Supplement: Supplementary file 1 [file foods-11-02820-s001.zip › foods-1821624-supplementary.pdf]

Supplementary Material

Contents:

Part 1. Captions to supplementary Tables

Table S1. The amino acids content of proteins from different parts of oyster (*Crassostrea hongkongensis*) (mg/g protein).

Notes: TAA, total amino acids; EAA, * essential amino acids; DAA, # delicious amino acids; NEAA, nonessential amino acids; HAA, Δ hydrophobic amino acids.

Part 2. Captions to supplementary Figure

Figure S1. Different tissue parts of oyster (*Crassostrea hongkongensis*).

Figure S2. Ultraviolet absorption spectra of proteins from different parts of oyster.

Notes: WSPV, WSPM, WSPG, and WSPA are water−soluble proteins of visceral mass, mantle adductor, respectively; SSPV, SSPM, SSPG, and SSPA are salt−soluble proteins of visceral mass, mantle adductor, respectively; ASPV, ASPM, ASPG, and ASPA are acid−soluble proteins of visceral mass, mantle adductor, respectively; ALSPV, ALSPM, ALSPG, and ALSPA are alkali−soluble proteins of visceral mass, mantle adductor, respectively.

Table S1 The amino acids content of proteins from different parts of oyster（*Crassostrea hongkongensis*）（mg/g protein）

Amino acids	WSP				SSP				ASP				ALSP			
	V	M	G	A	V	M	G	A	V	M	G	A	V	M	G	A
Asp#	141.73	115.02	109.16	96.58	115.32	118.85	99.28	102.40	115.38	118.32	92.91	106.72	114.04	113.98	95.04	99.66
Thr*	59.54	47.99	50.63	44.64	48.12	51.52	48.13	43.53	48.91	47.04	45.45	47.64	49.36	50.04	46.70	44.15
Ser	44.87	43.27	48.53	44.96	43.39	46.25	47.96	44.51	47.77	47.82	49.08	46.39	46.56	47.72	48.71	48.82
Glu#	149.28	178.95	124.15	158.70	179.42	175.64	130.81	190.77	166.81	167.71	123.38	186.57	154.71	144.71	124.67	155.01
ProΔ	44.03	25.70	39.02	31.14	25.77	26.49	35.72	22.59	27.04	29.87	50.32	26.74	38.31	41.85	43.19	69.65
Gly#Δ	55.77	32.70	43.53	38.13	32.78	32.06	42.60	25.98	38.80	36.43	82.92	31.34	52.16	60.23	60.14	133.24
Ala#Δ	59.12	68.65	42.24	52.42	68.83	63.52	46.29	61.29	63.19	65.25	39.71	63.31	56.43	55.44	39.92	46.80
Cys	4.53	4.72	10.48	4.29	5.22	3.66	10.23	2.74	3.92	3.93	6.99	3.86	1.77	3.09	3.39	3.42
Val*Δ	61.22	59.87	41.60	40.35	60.02	47.86	43.77	37.17	45.49	46.25	39.59	45.65	50.39	51.89	40.93	37.47
Met*	18.87	20.66	20.32	6.35	20.71	19.17	20.96	9.21	17.44	13.76	21.85	19.03	24.16	20.54	21.72	25.50
Ile*Δ	39.00	45.55	39.34	42.10	45.67	49.91	41.09	40.13	45.75	43.50	35.46	53.48	50.83	50.66	37.66	38.40
Leu*Δ	58.29	91.75	71.27	83.56	91.99	94.70	77.31	94.84	95.41	96.31	61.94	97.64	91.35	84.48	70.94	69.19
Tyr#	32.71	33.02	39.02	35.42	33.11	34.98	41.42	33.22	39.30	40.88	43.71	33.71	39.49	44.02	47.58	30.94
Phe*#Δ	39.84	34.98	43.37	37.01	35.07	36.01	40.92	28.72	34.63	34.98	37.21	38.06	48.92	50.66	43.57	36.38
Lys*	72.12	79.55	67.56	75.30	79.76	82.70	78.32	83.21	76.46	72.72	56.57	88.56	68.66	65.02	57.75	44.62
His	53.67	17.08	34.67	18.27	17.13	15.22	21.80	14.91	18.07	15.33	27.22	18.91	18.27	20.08	23.98	13.68
Trp*Δ	16.35	17.89	121.57	117.24	14.84	10.10	106.66	78.72	12.76	15.33	113.89	3.73	14.14	18.38	130.57	20.99
Arg	49.06	82.64	53.53	73.55	82.86	91.33	66.74	86.07	102.87	104.56	71.80	88.68	80.45	77.22	63.53	82.09
TAA	1000	1000	1000	1000	1000	1000	1000	1000	1000	1000	1000	1000	1000	1000	1000	1000
EAA/TAA (%)	36.52	39.82	45.57	44.65	39.62	39.20	45.72	41.55	37.68	36.99	41.20	39.38	39.78	39.17	44.98	31.67
EAA/NEAA (%)	57.54	66.18	83.71	80.68	65.61	64.47	84.21	71.09	60.47	58.70	70.06	64.96	66.06	64.38	81.77	46.35
DAA/TAA (%)	47.84	46.33	40.15	41.83	46.45	46.11	40.13	44.24	45.81	46.36	41.98	45.97	46.57	46.90	41.09	50.20
HAA/TAA (%)	37.36	37.71	44.20	44.19	37.50	36.07	43.43	38.94	36.31	36.79	46.10	36.00	40.25	41.36	46.69	45.21

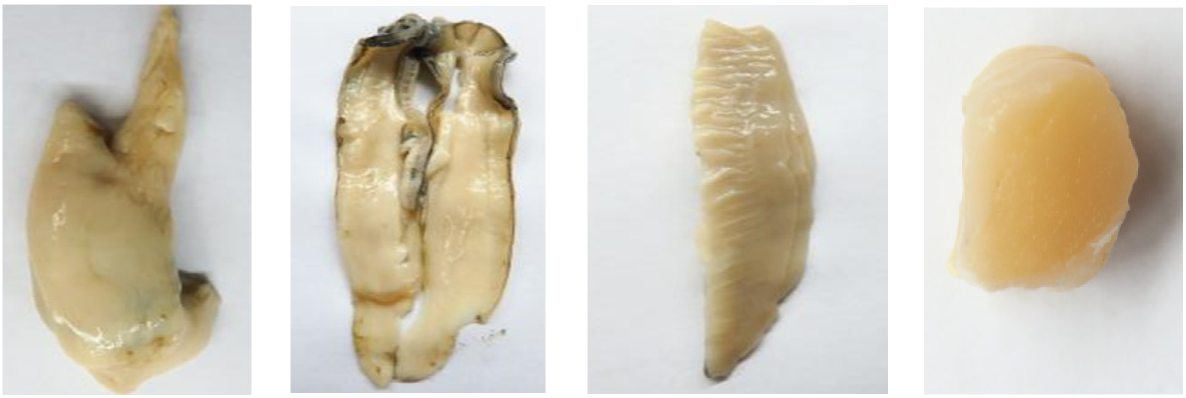

Visceral Mass Mantle Gill Adductor

Figure S1

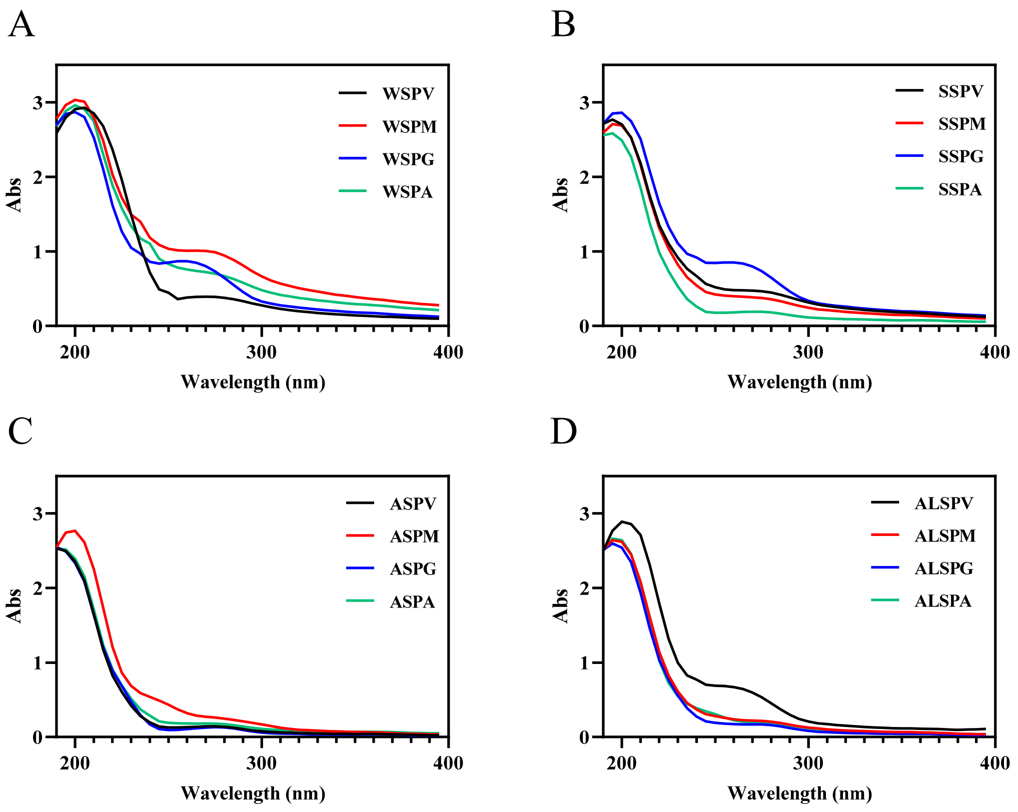
A
WSPV
WSPM
WSPG
WSPA
Abs
Wavelength (nm)
B
SSPV
SSPM
SSPG
SSPA
Abs
Wavelength (nm)
C
ASPV
ASPM
ASPG
ASPA
Abs
Wavelength (nm)
D
ALSPV
ALSPM
ALSPG
ALSPA
Abs
Wavelength (nm)

Figure S2
